# Effect of epigallocatechin-3-gallate on proliferation and phenotype maintenance in rabbit articular chondrocytes *in vitro*

**DOI:** 10.3892/etm.2014.2057

**Published:** 2014-11-10

**Authors:** HAOJIA HUANG, QIN LIU, LEI LIU, HUAYU WU, LI ZHENG

**Affiliations:** 1 Graduate School, Guangxi Medical University, Nanning, Guangxi 530021, P.R. China; 2 Research Center for Regenerative Medicine, Guangxi Medical University, Nanning, Guangxi 530021, P.R. China; 3 Medical and Scientific Research Center, Guangxi Medical University, Nanning, Guangxi 530021, P.R. China; 4 Department of Cell Biology and Genetics, School of Premedical Sciences, Guangxi Medical University, Nanning, Guangxi 530021, P.R. China

**Keywords:** epigallocatechin-3-gallate, pro-chondrogenic agent, chondrocyte, rabbit articular cartilage, dedifferentiation

## Abstract

In autologous chondrocyte implantation (ACI) to restore defective cartilage, limited cell numbers and dedifferentiation of chondrocytes are the major difficulties. An alternative is the use of growth factors, but their high cost and potential for tumorigenesis are major obstacles. To ensure successful ACI therapy, it is important to find an effective substitute pro-chondrogenic agent. Epigallocatechin-3-gallate (EGCG), one of the green tea catechins, has been widely investigated in studies of interleukin-1β-induced chondrocytes. In the present study, the effects of EGCG on rabbit articular chondrocytes were investigated through the examination of cell proliferation, morphology, glycosaminoglycan synthesis and cartilage-specific gene expression. The results showed that EGCG could effectively promote chondrocyte growth and enhance the secretion and synthesis of the cartilage extracellular matrix by upregulating expression levels of aggrecan, collagen II and Sox9 genes. Expression of the collagen I gene was downregulated, which showed that EGCG effectively inhibited the dedifferentiation of chondrocytes. Hypertrophy, which may lead to chondrocyte ossification, was also undetectable in the EGCG groups. In conclusion, the recommended dose of EGCG was found to be in the range of 5 to 20 μM, with the most marked response observed with 10 μM. The present study may provide a basis for the development of a novel agent as a substitute for growth factors in the treatment of articular cartilage defects.

## Introduction

Unlike other tissues, articular cartilage is avascular and has a poor healing potential following defects (1,2). Autologous chondrocyte implantation (ACI) is highly recommended for articular cartilage repair (3). During the process of ACI, the isolation of chondrocytes from the donor tissue and expansion of the cells *in vitro* are necessary; however, this approach is confronted with several problems, including the limited number of isolated chondrocytes and the loss of chondrocyte phenotypes. An alternative is the use of growth factors but their popularization and application in the clinic is limited for a number of reasons: i) Growth factors may induce the formation of osteophytes, resulting in the degeneration of articular cartilage (4); ii) tumorigenesis may occur (5–7); and iii) growth factors are generally expensive. The identification of another molecular substance to substitute for growth factors in the restoration of defects is, therefore, important.

The possible beneficial health effects of green tea have received considerable attention. The polyphenols in green tea, catechins, which include (−)-epigallocatechin-3-gallate (EGCG), (−)-epigallocatechin, (−)-epicatechin-3-gallate and (−)-epicatechin, are major constituents in brewed green tea (8). It is known that these green tea polyphenols are effective free radical scavengers and potent antioxidants (9,10), and there is considerable epidemiological evidence suggesting that there is an inverse correlation between green tea consumption and cancer development (11–14). Other polyphenols with strong antioxidant properties, found in foods or beverages such as tea, grape and turmeric, have shown both cancer chemopreventative and chemotherapeutic effects in numerous cell culture systems and animal tumor bioassays (15–17). Among various nutraceutical ingredients, EGCG, the most abundant and most active catechin derivative in green tea, is predominantly accountable for the biological effects of green tea (15).

EGCG is the most active and widely found polyphenol in green tea and is well known to be a primary contributor to the potential benefits of green tea to human health (18–20). The main mechanisms underlying the action of EGCG have been suggested to involve its potent antioxidant activity, which allows neuro- and cardioprotection (21,22). Other favored mechanisms entail the chemopreventative, anti-inflammatory, antithrombotic and cytoprotective effects of EGCG (23–25). The protective effect of extracts of EGCG on the metabolism of human chondrocytes in cartilage alteration has also been demonstrated (26,27). Given that EGCG has beneficial health effects, it was hypothesized that it could be a potential chondroprotective agent to replace growth factors when applied in ACI. In this study, the effect of EGCG on chondrocytes and their growth *in vitro* was investigated. Examination of the cell proliferation, morphology, glycosaminoglycan (GAG) synthesis and cartilage-specific gene expression following treatment with EGCG was performed. The present study may provide a basis for the development of a novel agent that can replace growth factors in the treatment of articular cartilage defects.

## Materials and methods

### Materials and chemicals

EGCG (purity ≥98%, high-performance liquid chromatography) was purchased from Shanghai Yuanye Bio-Technology Co., Ltd. (Shanghai, China) and stored at 4°C. Prior to the experiments, EGCG was dissolved in dimethylsulfoxide (Beijing Solarbio Science and Technology Co., Ltd., Beijing, China) and kept at −20°C until ready for use.

### Articular chondrocyte culture

Articular chondrocytes were harvested from knee joint cartilage slices of one-week-old New Zealand rabbits (Animal Center of Guangxi Medical University, Nanning, China) by enzymatic digestion. In brief, cartilage slices from two rabbits were dissociated enzymatically with 0.25% trypsin (Beijing Solarbio Science and Technology Co., Ltd.) for 30 min and then with 2 mg/ml collagenase type II (Gibco-BRL, Carlsbad, CA, USA) in α-modified Eagle’s medium (α-MEM; Gibco-BRL) for 3 h. Following centrifugation, the chondrocytes were resuspended. The cells were cultured with α-MEM containing 20% (v/v) fetal bovine serum (Gibco-BRL) and 1% (v/v) penicillin/streptomycin (Beijing Solarbio Science and Technology Co., Ltd.) in a 5% CO_2_ humidified incubator at 37°C with the culture medium replaced every other day after plating. Articular chondrocytes at passage 2, with a cell density of 2×10^4^/ml, were used for further studies. Cells were treated with taurine at a final concentration of 15, 30 and 60 μg/ml, and a group without taurine-treatment served as a control. This study was approved by the Institutional Ethical Committee of Guangxi Medical University (approval no 20140121).

### Cell proliferation analysis and biochemical assay

Subsequent to being cultured for 2, 4 and 6 days, the cells were washed three times with phosphate-buffered saline (PBS). Cell precipitations were collected following digestion with Proteinase K solution (Sigma, St Louis, MO, USA) for 16 h at 60°C. The DNA production was measured by a spectrofluorometer (UV-1700, Shimadzu Company, Kyoto, Japan) using Hoechst 33258 (Sigma) dye at 460 nm with the absorbance value of Hoechst 33258 dye alone as the baseline. The total secretion of GAG was quantified by absorbance value employing a 1,9-dimethylmethylene blue (Sigma) spectrophotometric assay at 525 nm with chondroitin sulfate as the standard sample. The total GAG secretion in each well was calculated according to the standard curve. The secretion of GAGs in each chondrocyte was normalized to the total DNA production of the chondrocytes, which indicated the biosynthetic activity of the cells in various culture media.

### Morphological examination

Subsequent to being cultured for 6 days, the cells were washed three times with PBS and fixed in 95% alcohol for 30 min. The cells were then washed with PBS solution and stained with a hematoxylin and eosin kit (Jiancheng Institute of Biotechnology, Nanjing, China). Finally, the cells were observed and photographed using an inverted phase contrast microscope (Axiovert200; Zeiss Corporation, Oberkochen, Germany).

### Reverse transcription-quantitative polymerase chain reaction (RT-qPCR) analysis

The gene expression of types I, II and X collagen, aggrecan and Sox9 was analyzed by RT-qPCR detection. Total RNA was sequentially extracted with an additional purification step employing an RNA isolation kit [Tiangen Biotech (Beijing) Co., Ltd., Beijing, China] according to the manufacturer’s instructions. An equal quantity of RNA (300 ng) was used as a template and reverse transcribed into cDNA using an RT kit (Fermentas, Burlington, ON, Canada), and then amplified using a SYBR Green Realtime PCR Master Mix kit (Roche Diagnostics, Mannheim, Germany) on a real-time fluorescence quantitative instrument (RealPlex 4; Eppendorf Corporation, New York, NY, USA). The primers (from Parkson Company, Shanghai, China) used for PCR are shown in (Table I). The dissociation curve of each primer pair was analyzed to confirm the primer specificity. Marker gene expression levels of the chondrocytes were analyzed by the 2^−ΔΔCT^ method using GAPDH as the internal control. Each sample was repeated three times for each gene.

### Statistical analysis

Data are presented as the mean ± standard deviation. Statistical significance was determined using one-way analysis of variance followed by Dunnett’s post hoc test. P<0.05 was considered to indicate a statistically significant difference.

## Results

### Cell proliferation

**Figure 1:**
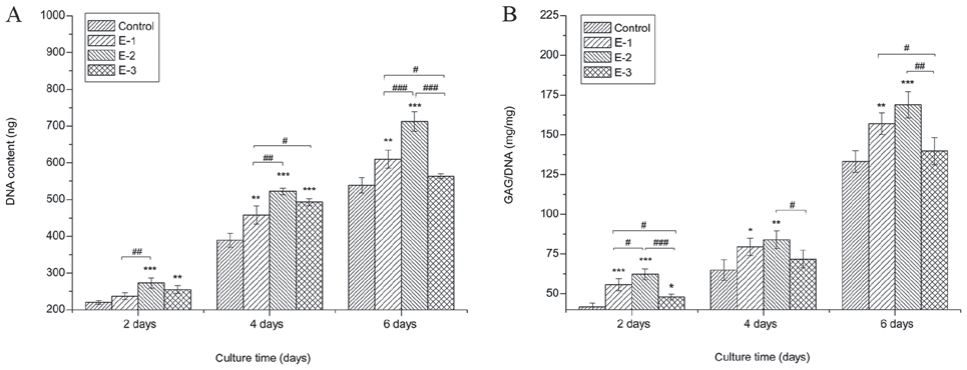
Quantification of cell proliferation by detection of DNA content, and matrix production by GAG analysis. (A) Proliferation of chondrocytes cultured *in vitro* with 0 μM (control), 5 μM (E-1), 10 μM (E-2) and 20 μM (E-3) epigallocatechin-3-gallate for 2, 4 and 6 days; (B) GAG content (mg) normalized to DNA content (mg). Data from four independent experiments were evaluated and the mean ± standard deviation is shown. ^*^ and ^#^ indicate P<0.05; ^**^ and ^##^ indicate P<0.01; ^***^ and ^###^ indicate P<0.001 compared with the control group at the same time point. GAG, glycosaminoglycan.

The proliferation of chondrocytes cultured with various concentrations of EGCG (0, 5, 10 and 20 μM) was detected by the DNA content measurements. Cells cultured with EGCG grew faster than those in the control group (P<0.05), as proved by distinctly higher DNA values than the control in the same culture period. Among the groups, the greatest cell proliferation was achieved with 10 μM EGCG. The results indicated that EGCG facilitated chondrocyte growth, particularly at the concentration of 10 μM (Fig. 1A).

### Secretion of GAGs

To determine if extracellular GAG production was affected by EGCG, biochemical assays were performed after 2, 4 and 6 days of culture. The results of intracellular GAG production following the treatment of chondrocytes with different concentrations of EGCG (Fig. 1B) showed that GAG production in culture media treated with EGCG was significantly improved over that in the control at the same time-point. In particular, EGCG at a concentration of 10 μM exhibited the strongest promotion of GAG synthesis among the three concentrations.

### Cell morphology

**Figure 2:**
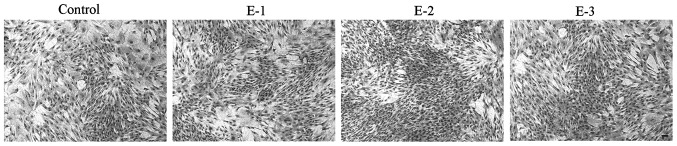
Hematoxylin and eosin staining images showing the morphology of chondrocytes cultured *in vitro* with 0 μM (control), 5 μM (E-1), 10 μM (E-2) and 20 μM (E-3) epigallocatechin-3-gallate for 6 days. Cell seeding density, 2×10^4^/ml; original magnification, ×100; scale bar = 200 μm.

The morphology of articular chondrocytes following treatment with EGCG at various concentrations (0, 5, 10 and 20 μM) is shown in Fig. 2. No evident difference in cell morphology was observed between cells with and without EGCG after 6 days in culture. Compared with the control, the chondrocytes in the presence of EGCG grew better and appeared to have a superior proliferation tendency with the gradually increasing days. At a concentration of 10 μM, EGCG could better facilitate the proliferation of chondrocytes than at other concentrations.

### Gene expression

**Figure 3:**
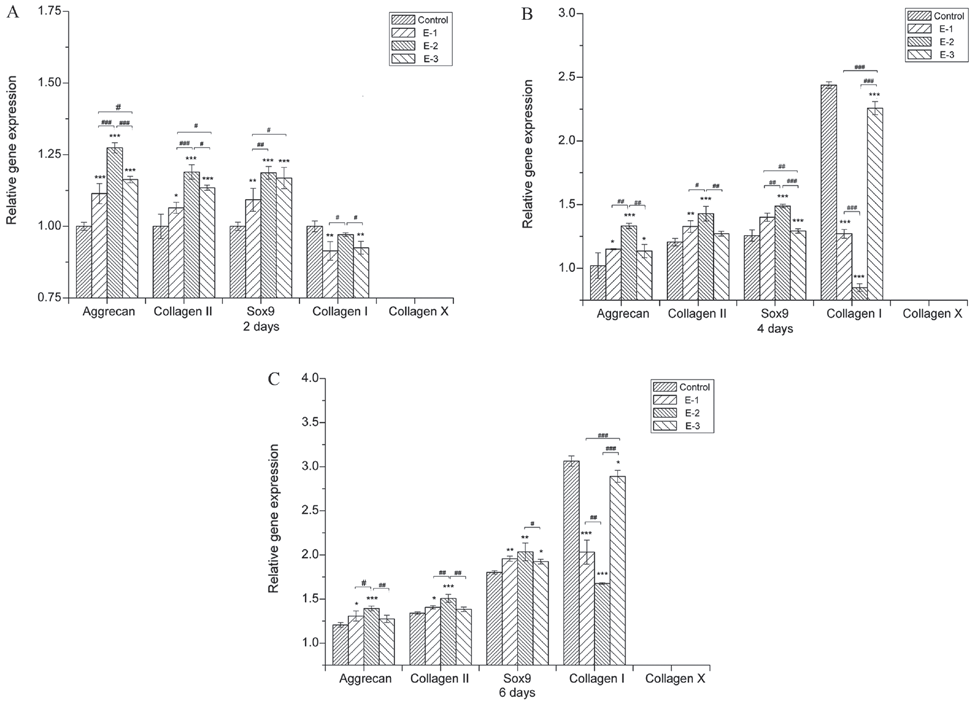
Quantitative comparison of extracellular matrix-related gene expression by reverse transcription-quantitative polymerase chain reaction. The chondrocytes were cultured with 0 μM (control), 5 μM (E-1), 10 μM (E-2) and 20 μM (E-3) EGCG for (A) 2, (B) 4 and (C) 6 days (n=3 for each experiment). The gene expression levels in the EGCG media relative to those in the control group were analyzed by the 2^−ΔΔCT^ method using GAPDH as the internal control. The data are presented as the mean ± standard deviation of three independent culture experiments. ^*^ and ^#^ indicate P<0.05; ^**^ and ^##^ indicate P<0.01; ^***^ and ^###^ indicate P<0.001 compared with the control group at the same time point. EGCG, epigallocatechin-3-gallate.

The effect of EGCG on the extracellular matrix (ECM) synthesis by chondrocytes was further examined through the gene expression of collagen I, collagen II, collagen X, Sox9 and aggrecan [a proteoglycan (PG) composed of GAGs] after 2, 4 and 6 days of culture. As shown in Fig. 3, cartilage-specific gene expression, such as aggrecan, collagen II and Sox9, was significantly enhanced by EGCG at concentrations of 5–20 μM with the 10 μM group showing the highest aggrecan, collagen II and Sox9 expression. The presence of EGCG upregulated aggrecan, collagen II and Sox9 expression, suggesting that EGCG either delayed or prevented the chondrocytes from dedifferentiating into a hypertrophic phenotype. At the same time, collagen X expression was downregulated in all groups, indicating that cell dedifferentiation and hypertrophy were not significant.

EGCG at various concentrations led to lower collagen I expression when compared with the control group subsequent to being in culture for 2, 4 and 6 days. In addition, the levels of collagen I in the 10 μM group were lower than those in other groups. These results further suggested that EGCG could inhibit the dedifferentiation of chondrocytes.

Concentrations of EGCG ranging from 5 to 20 μM therefore upregulated the synthesis of cartilage markers. Among the groups, EGCG at a concentration of 10 μM produced the highest aggrecan and collagen II expression, which was consistent with the results of GAG production (Fig. 1B).

## Discussion

Anti-inflammatory agents may provide anti-arthritic effects to facilitate the resolution of cartilage inflammation following injury. EGCG is reported to have a role in the treatment of osteoarthritis (26,28). The present study focused on the effects of EGCG on primary rabbit chondrocytes to demonstrate EGCG as a potential pro-chondrogenic agent that can replace growth factors in cell-based therapies for cartilage repair.

The present study showed that EGCG, which is a novel antioxidant, could well support the growth of chondrocytes. As demonstrated by cell proliferation assays and morphological examination, EGCG could significantly promote chondrocyte growth compared with the control. Furthermore, EGCG could markedly promote GAG deposition in chondrocytes, as shown by biochemical assay (Fig. 1B). PGs are important components of ECMs (29). For all PGs, GAGs constitute a major component of their molecular mass; furthermore, GAGs and a large number of water molecules generate expansion pressure and make the cartilage flexible, which plays an important role in maintaining cartilage load-bearing capacity (30). Consistent with the increase in GAG production, EGCG could upregulate the gene expression of cartilage-specific aggrecan, collagen II and Sox9 (Fig. 3). The chondrogenic transcription factor Sox9 plays a major role in an increased level of chondrogenesis (31,32), in particular activating co-expression with collagen type II (33–35). In addition, extensive gene therapy approaches using viral methods to overexpress Sox9 have resulted in marked improvements in the secretion of cartilaginous matrix by articular chondrocytes, bone marrow-derived stem cells and nucleus pulposus cells (36–38). These data indicated that EGCG could facilitate chondrocyte proliferation and stimulate exuberant cartilage matrix secretion.

The expression of collagen type I, which marks the dedifferentiation of chondrocytes, was effectively inhibited by EGCG. Dedifferentiation occurs when the differentiated phenotype of chondrocytes, primarily composed of type II collagen and cartilage-specific PGs, is lost and replaced by a complex collagen phenotype consisting of a majority of type I collagen and a low level of PG synthesis (39–41). Furthermore, collagen type X, which is specifically associated with hypertrophic chondrocytes and precedes the onset of endochondral ossification (42), was nearly undetectable in the EGCG groups, indicating that the hypertrophy of chondrocytes would not be induced by EGCG. As a consequence, the decreasing collagen I expression and the inconspicuous expression of collagen X could suggest that EGCG prevents the dedifferentiation and hypertrophy of chondrocytes.

EGCG, which is the ester of epigallocatechin and gallic acid, has been found to inhibit the degradation of human cartilage PG and type II collagen, and selectively inhibits a disintegrin and metalloproteinase with thrombospondin motifs (ADAMTS)-1, ADAMTS-4, and ADAMTS-5 (43,44). Further research has revealed that EGCG ameliorates the interleukin-1β-mediated suppression of transforming growth factor-β synthesis, and enhances type II collagen and aggrecan core protein synthesis in human articular chondrocytes (45).

With regard to the recommended dose of EGCG, the present results demonstrated that DNA synthesis of rabbit articular chondrocytes was increased in a dose-dependent manner when chondrocytes were cultured in medium containing EGCG at concentrations of 5–20 μM; EGCG at 10 μM could support the strongest cell proliferation and stimulate the greatest matrix secretion.

## Figures and Tables

**Table I:** Primer sequences used in the quantitative polymerase chain reaction experiments.

mRNA	Forward primer	Reverse primer
GAPDH	5′-CTATAAATTGAGCCCGCAGC-3′	5′-ACCAAATCCGTTGACTCCG-3′
Aggrecan	5′-CTACACGCTACACCCTCGAC-3′	5′-ACGTCCTCACACCAGGAAAC-3′
Type I collagen	5′-GTTCAGCTTTGTGGACCTCCG-3′	5′-GCAGTTCTTGGTCTCGTCAC-3′
Type II collagen	5′-AAGCTGGTGAGAAGGGACTG-3′	5′-GGAAACCTCGTTCACCCCTG-3′
Type X collagen	5′-CGCTGAACGATACCAAATGCC-3′	5′-TTCCCTACAGCTGATGGTCC-3′
Sox9	5′-AAGCTCTGGAGACTTCTGAACG-3′	5′-CGTTCTTCACCGACTTCCTCC-3′
